# The Pathology of Asylum Dysentery

**Published:** 1904-05-07

**Authors:** 


### THE PATHOLOGY OF ASYLUM DYSENTERY.

Not the least of the many anxieties accruing to medical superintendents of hospitals for the insane is the recurrence year after year, and despite of every hygienic endeavour, of acute colitis among the patients at these institutions. Hitherto the etiology of the illness has remained unsolved and rational attempts towards its prevention have not unnaturally met with an incomplete measure of success. There seems to be, however, some prospect of obtaining better results from prophyllaxis in the near future; at any rate, these may be expected if more extended investigation should prove, what appears to be the case from Dr. Eyre's[1] researches that asylum dysentery in this country is due to a member of the group of organisms typified by bacillus dysenteriæ. A similar bacillus has already been demonstrated to be associated with epidemic diarrhœa occurring in asylums for the insane in America, so that there is already a good deal of positive evidence in favour of Dr. Mott's contention that tropical dysentery and asylum dysentery are terms for one and the same disease.

Dr. Eyre recently carried out examinations of material obtained from cases of epidemic colitis which occurred last year in one of the London county asylums, and from several of the specimens he was able to isolate the bacillus dysenteriæ. As Dr. Eyre points out, more extended inquiry must be effected before any definite generalisation as to the etiology of asylum dysentery can be made. In the meantime it is possible to put forward the following propositions:—(1) That a bacillus, identical with the B. dysenteriæ, described by Shiga as the specific cause of acute dysentery in Japan, can be isolated from the stools of many cases of asylum dysentery. (2) That the blood serum of some of these cases of asylum dysentery possesses a specific agglutinative action when tested against B. dysenteriæ isolated from the stools of other similar cases, and also against other strains of B. dysenteriæ isolated from cases of dysentery in tropical countries. (3) That in order to detect the presence of B. dysenteriæ the stools must be examined when fresh. (4) That post-mortem material must be collected and examined as soon after death as possible, as after a few hours' delay, even in quite acute cases, B. coli becomes the predominant organism in the intestinal canal. (5) That under these conditions (3 and 4) the isolation of B. dysenteriæ from the stools of patients suffering from acute asylum dysentery is comparatively easy when those stools are, to the naked eye, typically dysenteric. (6) That in cases of chronic asylum dysentery B. dysenteriæ, if present, is so outnumbered by B. coli and other intestinal saprophytes as to render its isolation a matter of extreme difficulty.

[1] Brit. Med. Jour., April 30.

